# Mutations in *RPS19* may affect ribosome function and biogenesis in Diamond Blackfan anemia

**DOI:** 10.1002/2211-5463.13444

**Published:** 2022-06-06

**Authors:** Disha‐Gajanan Hiregange, Andre Rivalta, Ada Yonath, Ella Zimmerman, Anat Bashan, Hagith Yonath

**Affiliations:** ^1^ The Department of Chemical and Structural Biology Weizmann Institute of Science Rehovot Israel; ^2^ Sackler School of Medicine Tel Aviv University Tel Aviv Israel; ^3^ Internal Medicine A Sheba Medical Center Ramat Gan Israel

**Keywords:** DBA, eS19, genetic mutations, ribosomes, ribosomopathies, *RPS19*

## Abstract

Ribosomes, the cellular organelles translating the genetic code to proteins, are assemblies of RNA chains and many proteins (RPs) arranged in precise fine‐tuned interwoven structures. Mutated ribosomal genes cause ribosomopathies, including Diamond Blackfan anemia (DBA, a rare heterogeneous red‐cell aplasia connected to ribosome malfunction) or failed biogenesis. Combined bioinformatical, structural, and predictive analyses of potential consequences of possibly expressed mutations in eS19, the protein product of the highly mutated *RPS19,* suggest that mutations in its exposed surface could alter its positioning during assembly and consequently prevent biogenesis, implying a natural selective strategy to avoid malfunctions in ribosome assembly. A search for *RPS19* pseudogenes indicated > 90% sequence identity with the wild‐type, hinting at its expression in cases of absent or truncated gene products.

## Graphical abstract

Diamond Blackfan anemia (DBA) is a rare congenital intrinsic erythroid hypoplasia, associated with mutated ribosomal genes. Combined bioinformatical, structural, and predictive analyses of potential consequences of expressed mutations in eS19, the protein product of the highest mutated *RPS19,* suggest that its surface‐exposed mutations could alter its positioning and consequently interfere with ribosome biogenesis, implying a natural selective strategy to avoid malfunctioning ribosomes.

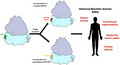

**Abbreviations**
RP: ribosomal proteins
rRNA: ribosomal RNA
DBA: Diamond Blackfan anemia
NMD: nonsense‐mediated mRNA decay

Ribosomes are extremely efficient molecular machines that accurately translate the genetic code into proteins in all living cells. In eukaryotes, the functional ribosomes are comprised of long rRNA chains containing ~ 6880 nucleotides and ~ 80 different RPs, which are arranged precisely [1, 2, 3] in two interacting unequal subunits (called 40S and 60S, according to their sedimentation coefficients). Ribosomes' performance is accomplished by a highly correlated intricate mechanism, enabled by cooperative contributions of their various components and interactions with non‐ribosomal cellular entities.

Typically, ribosome's biogenesis proceeds smoothly and efficiently [4, 5, 6, 7], although it requires substantial intracellular molecular trafficking, delicate cooperation between the ribosomal components, and specific interactions with cellular assembly, activation, and finalization factors [8, 9, 10, 11, 12, 13, 14, 15, 16, 17, 18, 19, 20]. The RPs play a significant role in coordinating the maturation of the ribosome [21, 22] and, together with natural or modified rRNA bases (e.g., the modified rRNA base 1248 pseudouridine, m1acp3Ψ), they are collectively implicated in maintaining ribosome biogenesis and in regulating its function [5, 23, 24, 25, 26, 27, 28].

Ribosomopathies, a collection of genetic diseases, are predisposition syndromes associated mainly with either impaired ribosome biogenesis or ribosome dysfunction [29, 30, 31, 32, 33, 34, 35, 36, 37, 38, 39, 40, 41, 42]. Mutations in genes coding for ribosomal proteins have been implicated in several congenital syndromes belonging to a heterogeneous group of disorders [40, 41, 42, 43] that share malfunctioning bone marrow, linked to blood impediments directly or via issues concerning heme export [44]. Among them, some have been associated with various physical abnormalities, such as cleft lip and/or palate and cardiac defects [45, 46, 47]. Sequencing exomes of affected individuals identified mutations in different genes linked to impaired ribosome biogenesis and decreased translational efficiency [48, 49, 50, 51] or defective mRNA translation [52].

Diamond Blackfan anemia (DBA), a rare congenital intrinsic erythroid hypoplasia, was discovered in 1936 [53] in infants and children and categorized in 1937 as congenital hyperplastic anemia [54]. Its clinical aspects were described in the 1960s [55] and the 1970s [56]. In the post human genome era, DBA was identified as the first human ribosomopathy [57], and in 2006, it was considered as a paradigm for a ribosome‐based disease [58]. In 2008, a database for DBA mutated ribosomal genes was constructed [46], further updated in 2010 [59]. Similar to several other ribosomopathies, DBA is connected to the tumor suppressor gene *TP53*, which plays a central role in controlling ribosome function under stress [30, 60, 61, 62] and provides a surveillance mechanism for ribosomal function. The classical DBA presentation is a significant red cell aplasia in young infants, with congenital malformations in about 50% of the patients [47, 55, 63, 64]. In recent years, milder ‘non‐classical’ cases with less distinct phenotypes have been identified. Currently, bone marrow transplantation extends patients' survival [65, 66].

Molecular pathogenesis studies showed that approximately half of all known DBA cases are attributed to mutations in the pre‐rRNA‐processing protein TSR2 [67, 68, 69, 70, 71, 72, 73, 74, 75, 76] and RP genes, primarily, but not exclusively, those of the small ribosomal 40S subunit. Impressively, despite the wealth of DBA‐associated mutated genes, DBA is linked mainly to mutations in *RPS19*, the first DBA ribosomal mutated gene to be discovered [69, 77, 78, 79, 80, 81, 82, 83, 84, 85, 86]. These mutations, which result in hematopoietic and developmental abnormalities [40, 59, 87, 88, 89, 90, 91, 92, 93, 94], account for 25% of all DBA patients [10, 79, 82] and illustrate the significance of eS19, the protein coded by the *RPS19* gene. In addition to eS19 involvement in ribosome biogenesis [95, 96], it plays a role in cellular regulation in humans. Some of these mutations may have a dominant negative effect (as was shown in mice [97]) by binding to its own mRNA [98], presumably by a similar mechanism that is exploited by other ribosomal proteins [99, 100, 101, 102, 103].

The remarkable similarity between the 3D structures of eS19 within the functionally active human ribosome, and that of isolated *Pyrococcus abyssi*, which shares only 36% sequence identity and 57% sequence similarity [104], hints at the significance of its 3D structure.

Sixty‐four different mutations in *RPS19* have been clinically identified in DBA patients despite the relatively short length of its coded protein eS19 (∼ 145 amino acids, depending on the species). These are spread throughout the protein and were shown to be connected to several pathologies [105]. Commonly, mutated *RPS19* is linked to dysregulation of deltaNp63 and p53 [40], defects in 18S ribosomal RNA synthesis, assembly of the small ribosomal subunit, ribosome maturation [106, 107, 108], and increased proteasome activity [109, 110].

Here, we describe our studies on the highly mutated gene *RPS19* and its coded protein, eS19. We performed structural, biophysical, mutational, and genomic comparative analyses of the potential outcomes of eS19 mutations by examining its structural and interactions patterns, as observed within the 3D structure of the human ribosome. Our results shed light on the natural response to the type of the mutation and the consequent expected implications on ribosome biogenesis or ribosome function. Furthermore, our analyses indicate the existence of an ingenious natural selection mechanism to avoid the disturbance of a ribosome malfunction by hindering the biogenesis of ribosomes with a mutated functional site, despite the risk that it may lead to a significant reduction in the ribosome level. In parallel, nonsense‐mediated mRNA degradation (NMD) analysis [111] indicated that a small number of predicted mutations in *RPS19* could diminish eS19 transcription and translation. Subsequently, we identified a pseudogene that highly resembles the natural *RPS19* gene, thus may replace it under specific circumstances, such as heavily truncated or entirely deleted protein.

## Materials and methods

Information about the mutations was extracted from published reports [46, 59, 88, 89, 90, 91, 92, 93]. The PDB files with IDs 4UG0 [2], 6G4W [15], and 6G53 [14] were the sources for the structural details of the fully and partially assembled human ribosome that were used for the various analyses. Coordinates of the different maturation states of the partially assembled large ribosomal subunit were taken from Ameismeier et al. [15]. The distances reported in Table S2 were calculated using chimerax [112, 113]. The PDB IDs of the various assembly states are shown in each sheet; in PDB 6G4W, the side of eS19 facing the assembly factor *RRP12*, and *RRP12* itself is less well resolved; hence, the distances were calculated by substituting the original eS19 chain with the one from 4UG0.

COOT [114] and ucsf chimera [115] were used for generating the atomic models. The mutations were mapped on the models by using ucsf chimera.

*RPS19* genes, pseudogenes, and mRNA sequences for the mutation analysis were extracted from NCBI (gene ID 6223, transcript ID NM_001022.4).

Pseudogene sequences were taken from the NCBI RefSeq track [116] in the UCSC Genome Browser (Human Genome version hg38) [117] and translated to amino acids using Expasy Translate (https://web.expasy.org/translate/) [118].

The predictions of the amino acid sequences of the mutated proteins were performed using Expasy Translate (https://web.expasy.org/translate/). Nonsense‐mediated mRNA degradation prediction tool [119] was used to predict which mutated transcript variants escaped NMD. The NMD for point mutations (missense/nonsense) were predicted using the 50–55 nucleotide rule. EMBOSS NEEDLE [120] was used to calculate the percentage identity, similarity, and gaps between each pseudogene and *RPS19* wild‐type. Sequence alignments were performed with clustalw [121] and visualized in jalview [122]. SWISS‐MODEL [123, 124, 125, 126, 127] was used to predict mutated eS19 with 4UG0 chosen as a template. ucsf chimerax was used to superimpose the wild‐type eS19 and mutated eS19 models that SWISS‐MODEL generated. Potential H‐bonds were calculated using the built‐in tool in uscf chimerax.

## Results

### Distribution of RPs mutations in DBA

**Fig. 1:**
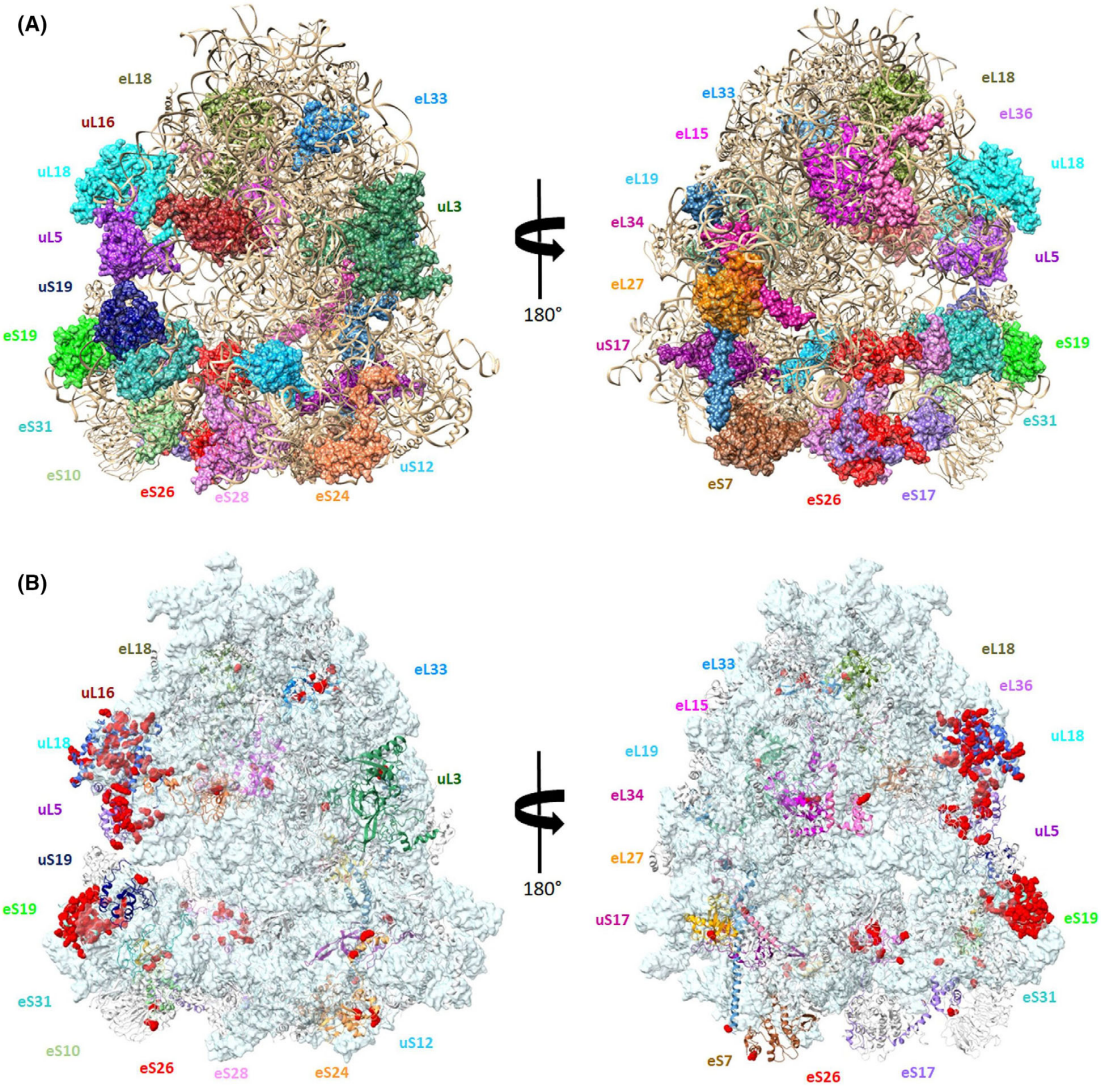
(A) Surface representations of the human ribosome (PDB ID 4UG0), showing all ribosomal proteins whose genes were found mutated, in variable colors. The rRNA and the unaffected proteins are shown as light brown ribbons. The two images are 180° rotated with respect to each other. (B) Surface representations of the human ribosome (PDB ID 4UG0), showing that all mutations in the ribosomal proteins are exposed at the surface, and many of them around the 60S central protuberance and the 40S head. [Colour figure can be viewed at wileyonlinelibrary.com]

To seek insights into how the mutations that cause DBA may influence ribosomal biogenesis and/or functionality, we imposed all of the predictable mutations in the RPs on the human ribosome structure [1, 2, 3]. We noticed that the predictable mutations are distributed throughout the ribosome, mostly on or in proximity to the ribosome surface (Fig. 1). By placing the DBA mutation sites on the human ribosome structure, we gained insights into how such modifications may influence ribosome functionality and thus may reveal some aspects of cellular malfunctioning. We pursued comparative structural and genomic analyses related to the expected mutations at the RP level and their expected implications in the contexts of (a) ribosomal biogenesis and (b) the functionality of the ribosome. We studied the anticipated locations of each previously described DBA mutation within the RPs, based on the positions of the related features in the human ribosome structure (PDBID 4UG0) [2]. Then, we examined the distribution of viable DBA mutated ribosomal proteins within the human ribosome for selecting those suitable for predictive evaluation of the expected interactions with their rRNA surroundings.

Specifically, we focused on *RPS19*, and its encoded small ribosomal subunit protein, eS19, owing to the vast number of mutations identified in it in the DBA patients [10, 79, 82]. We expected that some of these mutations would be involved in the ribosome assembly as, in all states of the pre‐assembled ribosome, it is located in close proximity to the surface of the pre‐small subunit [14, 15, 128].

### Structural implications of RPS19 mutations in DBA

**Fig. 2:**
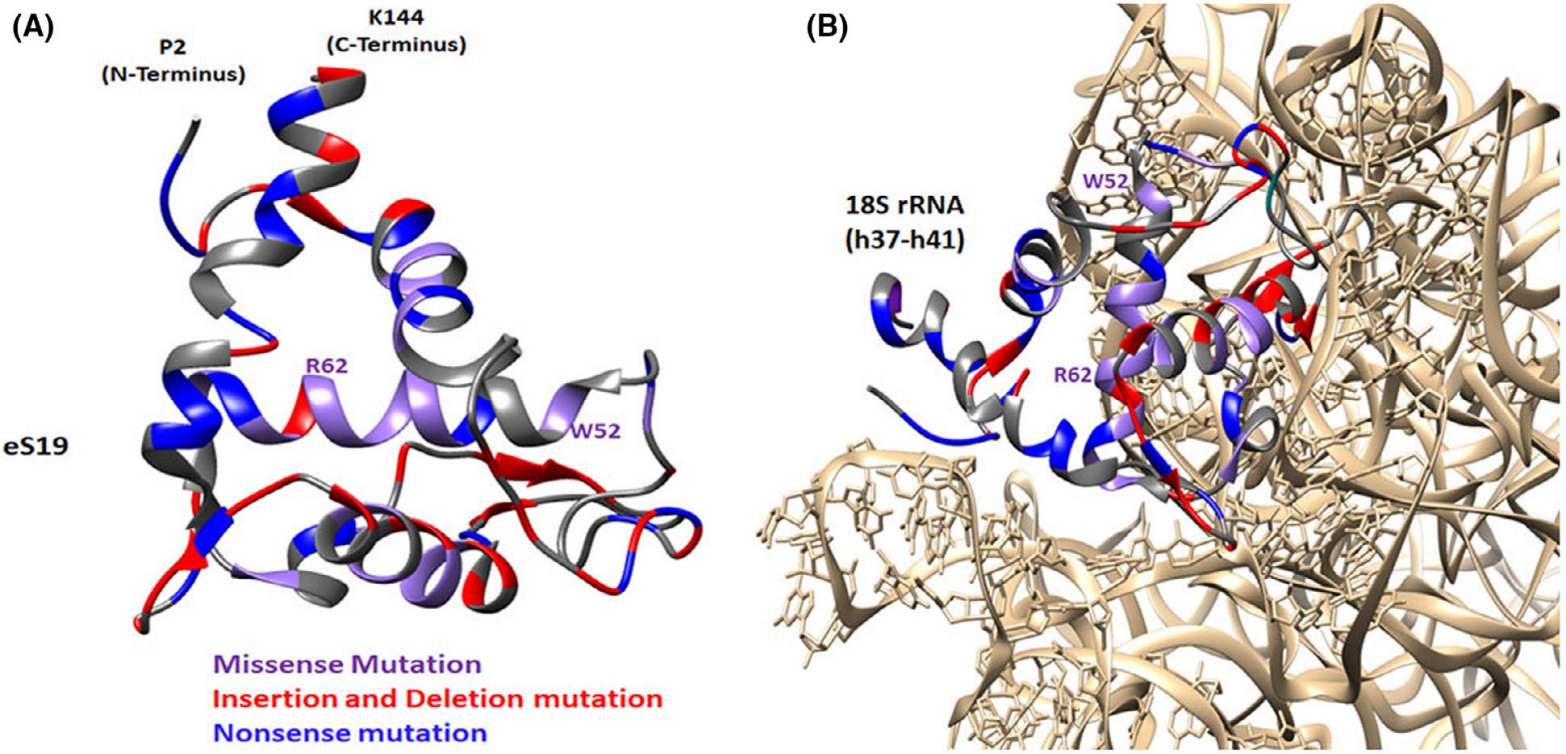
Expected Diamond Blackfan anemia (DBA) mutations in eS19 mapped on its 3D structure within the human ribosome. (A) The structure of the human eS19. (B) Zoomed view of the region of 18S rRNA that is interacting or located in close proximity to mutated amino acids of eS19. The 18S rRNA is shown in light brown. The rRNA bases interacting or located close to eS19 (1365–1595, i.e., h37–h41) are shown using atoms representation. eS19 residues affected by insertion/deletion mutations, nonsense mutations, and missense mutations are shown in red, blue, and purple, respectively. The mutation sites were taken from previous reports [46, 59, 88, 89, 90, 91, 92, 93] of DBA patients' data. [Colour figure can be viewed at wileyonlinelibrary.com]

To assess the various consequences of the *RPS19* mutations on its protein product, eS19, we mapped the positions of all known mutations on the eS19 3D structure within the human ribosome. We marked them according to their types, namely missense, nonsense, insertion, and deletions mutations (Fig. 2). As seen, the missense and nonsense mutations tend to cluster in the helical regions, whereas the insertions and deletions are located in loops or the less structured termini, which are less expected to cause substantial structural alterations but may be needed for interactions with the proximate rRNA.

**Fig. 3:**
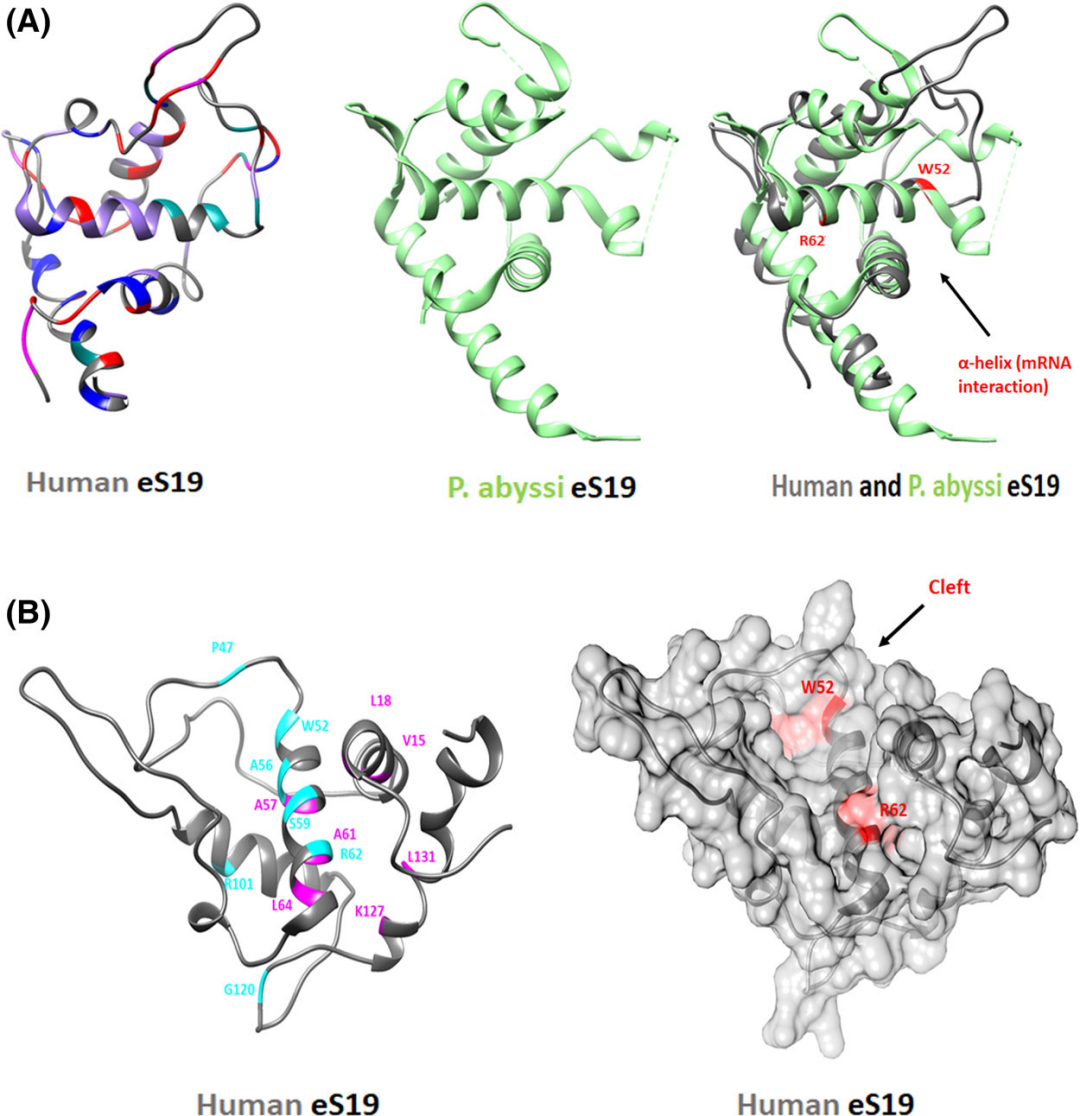
Structural similarity of human and the archaeal *Pyrococcus abyssi* protein eS19 [104], showing the cleft and main helix that seem to be involved in mRNA binding. (A) Left: The structures of human eS19 (PDBID 4UG0), in gray and colored mutations as in Fig. 2; middle: The structure of *P. abyssi* eS19 [104] (PDBID 2V7F). Note the partial lack of the observation of the flexible regions, or their modified structure, in the isolated protein vs. the ribosome‐incorporated protein; right: Overlay of both structures of eS19, of *P. abyssi* in green, and in gray within the human ribosome, on which residues W52 and R62 that are interacting with eS19 own mRNA [98] are marked. (B) Assumed mutated amino acids are marked as class I (pink) and class II (blue) on the structure of the human eS19, according to [104]. (C) Space‐filling surface representation of eS19 (gray). Amino acids W52 and R62 are marked in pink. The arrow points at the mRNA binding cleft. [Colour figure can be viewed at wileyonlinelibrary.com]

In addition, we examined the regions of the rRNA that interact with, or are located in close proximity to, eS19 (i.e., nucleotides 1365–1595, comprising helices h37‐h41). These rRNA residues may be affected by structural changes in eS19 upon mutations and consequently, should modify the ribosome structure and either affect ribosome biogenesis or intervene with ribosome functional activity. Our analysis was based on (a) the notion that several *RPS19* mutations could lead to impaired ribosome assembly and (b) the high conservation of eS19 structure (Fig. 3). Practically, this analysis was an attempt to structurally analyze the fate of the various mutations, namely to predict which mutations can be connected to failed biogenesis and which could be incorporated into assembled ribosomes and then be involved in ribosome malfunctioning.

**Fig. 4:**
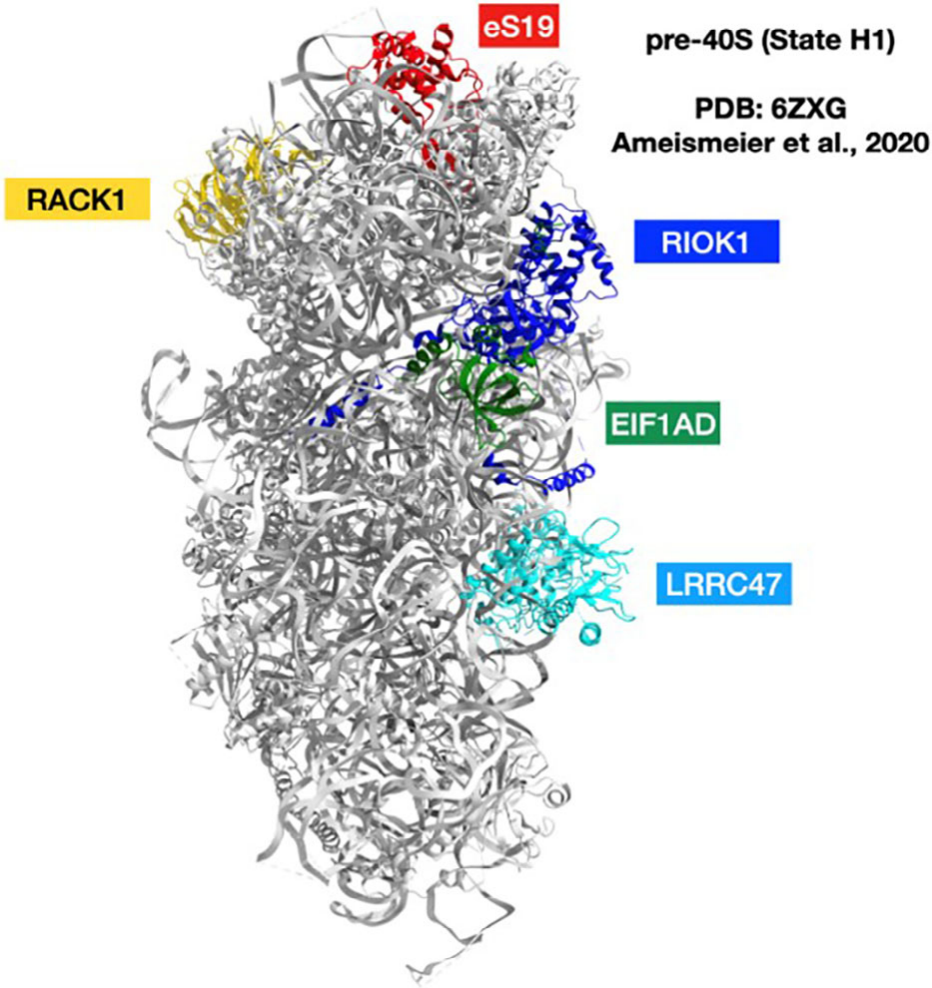
eS19 does not interact with any structurally studied assembly factors [14, 15]. [Colour figure can be viewed at wileyonlinelibrary.com]

Furthermore, based on the accumulated knowledge of ribosomal structure/function relationships, we assumed that RP mutations are likely to trigger structural modifications in their surroundings, which may generate additional structural modification in the rRNA or the RPs of the second or third shells around the mutations, and these may even propagate further and trigger additional modifications. For this analysis, we (a) classified the distribution of *RPS19* mutations according to their structural motifs (Table S1) (b) calculated the distances of all eS19 atoms to their neighborhood in the mature 40S, as well as in its various known assembly intermediate states of the creation of pre‐40S [14, 15] (c) identified interatomic ‘contacts’ by selecting all of the eS19 atoms whose distances to their neighbors are ≤ 4 Å (Table S2). We assumed that mutations of these atoms have a higher chance to influence the profile of the protein's interactions, which consequently may obstruct incorporation into the assembling ribosome and hamper the entire ribosome maturation. To assess whether the DBA mutations in eS19 affect or disrupt the rRNA environment during the small subunit (pre‐40S) maturation, we compared the different pre‐40S stages of wild‐type eS19 [14] and of the predicted mutated eS19 (Fig. S2). Following this, we inspected the potential H‐bonds that may be formed between eS19 and the surrounding environment. We analyzed the five states (A–E) that have been identified [14] and noticed that State D and State E had virtually identical H‐bonds. Hence, we considered only states A–D. For each state, to predict the H‐bonds of the mutated eS19, we replaced the existing eS19 chain with the mutated eS19 models that were generated by SWISS‐MODEL [123, 124, 125, 126, 127] and found that, as for these regions in native eS19, its expected mutants were not involved in any direct interaction with the non‐ribosomal factors, during the maturation process. As the natural H‐bonds are mostly between eS19 and the surrounding rRNA, the missense mutation should disrupt them, hence supporting the notion that some RP mutations in DBA patients might affect the ribosome maturation and assembly. Thus, by analyzing the interactions of eS19 with its rRNA vicinity in the assembly of 40S particle (Table S2), we added a new dimension to the evaluation of possible contributions of eS19 mutants to DBA. As the termini and internal loops are supposed to be relatively flexible and less structured (Fig. 3), intuitively, we expected that these should create a variety of positive and negative contacts with their surroundings during assembly. Indeed, we found that a few amino acids of these regions do interact with their neighboring rRNA or other RPs throughout the maturation process, but many of them do not (e.g., among the loop of residues 114–116, only residue 115 reaches their neighbors in a few stages). Similarly, in contrast to our initial thought, not even a single contact between eS19 and any assembly factors was found (Fig. 4).

It was shown that eS19 binds to its own mRNA in a fashion likely connected to a regulation strategy [98]. The highly conserved region involved in this regulation mechanism includes an α‐helix composed of residues 52–67, located at a rather exposed central part of the protein, on its polar face. In the DBA mutation database, the respective genomic region was shown to contain a large number of missense mutations in the exposed residues W52, R56, S59, and R62, and hence, it is called the ‘eS19 mutation hot spot’ [104]. Importantly, we found that residue R62, a part of this α‐helix, which is exposed on the surface of the mature ribosomes, is heavily involved in contacts with the neighboring rRNA during all assembly states [14, 15] (e.g., up to 45 contacts 2.9–4 Å with nucleotides C1542 and U1543, in a single assembly state). This unexpected finding seems to show that owing to the location of R62, almost all of its mutations should modify, disturb, or eliminate its contacts with its neighboring rRNA. These disturbances and the consequent non‐native contacts should harm the correct incorporation of the mutated eS19 into the pre‐40S particle, which should interfere with the creation of the 40S particle, thus perturbing the delicate biogenesis process and resulting in partially assembled ribosomal small subunits. In this way, we revealed a natural procedure to prevent the formation of functionally failing ribosomes as, if incorporated, its mutations are bound to interfere with proper ribosomal function.

### Mutation analysis of the links between ribosome biogenesis and maturation

**Fig. 5:**
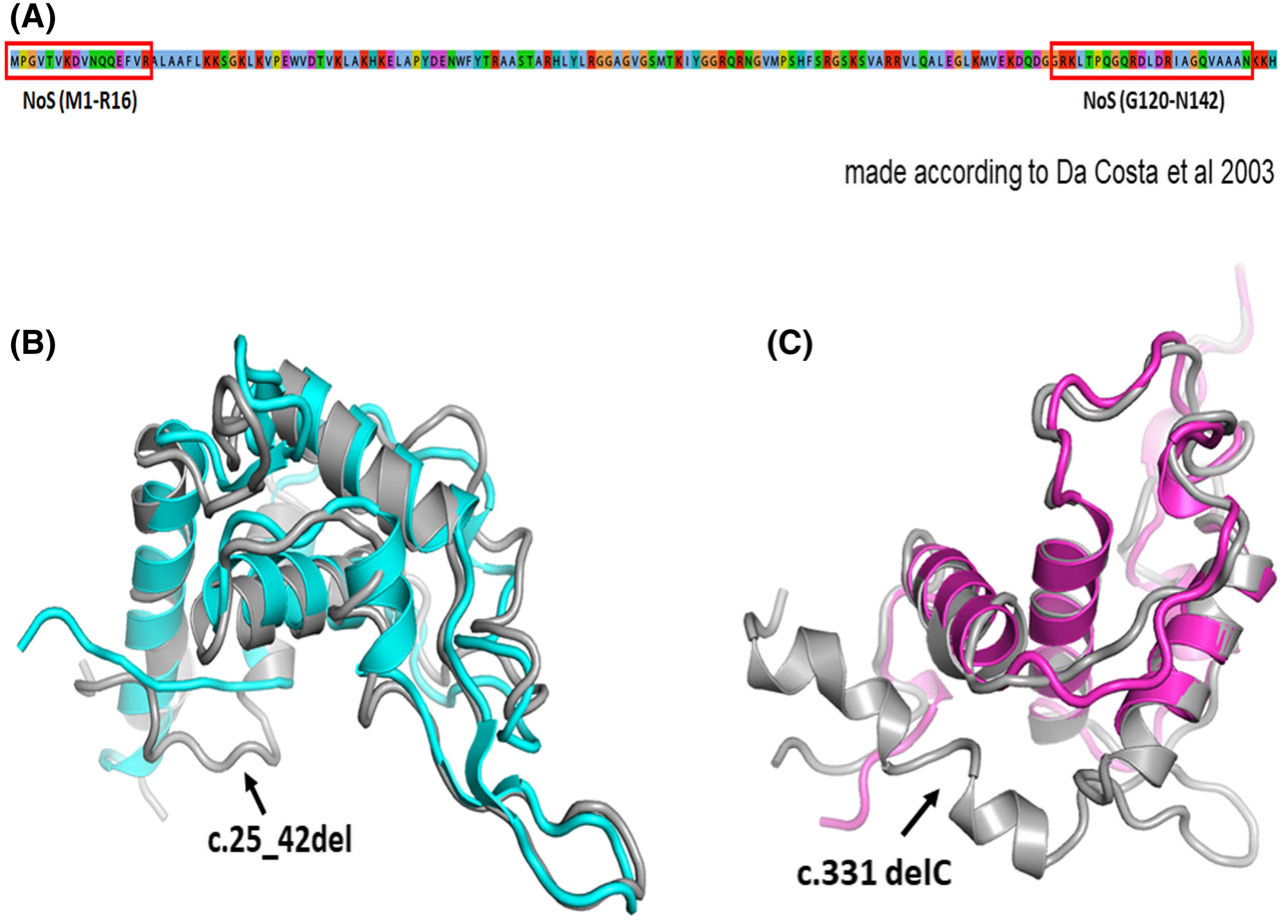
RPS19 insertion and deletion mutations affect eS19 length in Diamond Blackfan anemia. (A) Amino acid sequence of eS19 with nucleolar localization signal marked in a red box. (B) Structural comparison of predicted mutated eS19 with c.25_42del mutation (cyan) and wild‐type eS19 (gray). (C) Structural comparison of predicted mutated eS19 with c.331delC mutation (purple) and wild‐type eS19 (gray). [Colour figure can be viewed at wileyonlinelibrary.com]

We carried out an extensive structural and comparative analysis of the expected consequence of the genomic modifications and their diverse phenotypic implications. Prediction of the structure of the mutated eS19 using SWISS‐MODEL and its comparison with the structure of the wild‐type eS19 raised the possibility that the missense mutations might disrupt the neighboring rRNA environment (Fig. 2 and Fig. S1). In addition, our predicted structure of the eS19 with insertion and deletion mutations revealed that most of these DBA mutations would alter the length of the C terminus or its conformation (Fig. 5B,C). This might affect the protein localization in the nucleolus and/or hinder its role in ribosome biogenesis (Fig. 5).

### Genomic analysis of eS19 mutations in DBA and its transcripts

We mapped the detailed distribution of the known mutations on the exons of *RPS19* [88, 94] (Fig. S3) and calculated their expected influence on the lengths of the expressed proteins (Fig. S3). We also classified the *RPS19* mutations as per the distribution on the structural motifs of its protein product eS19 (Table S1). Our calculations and predictions revealed that mutations of the *RPS19* gene, including frameshift and nonsense, could result in a shorter eS19 (Fig. S4). These shorter proteins may still be incorporated into the ribosome and allow its function. An example is the C‐terminal tail, which extends into the rRNA environment.

**Fig. 6:**
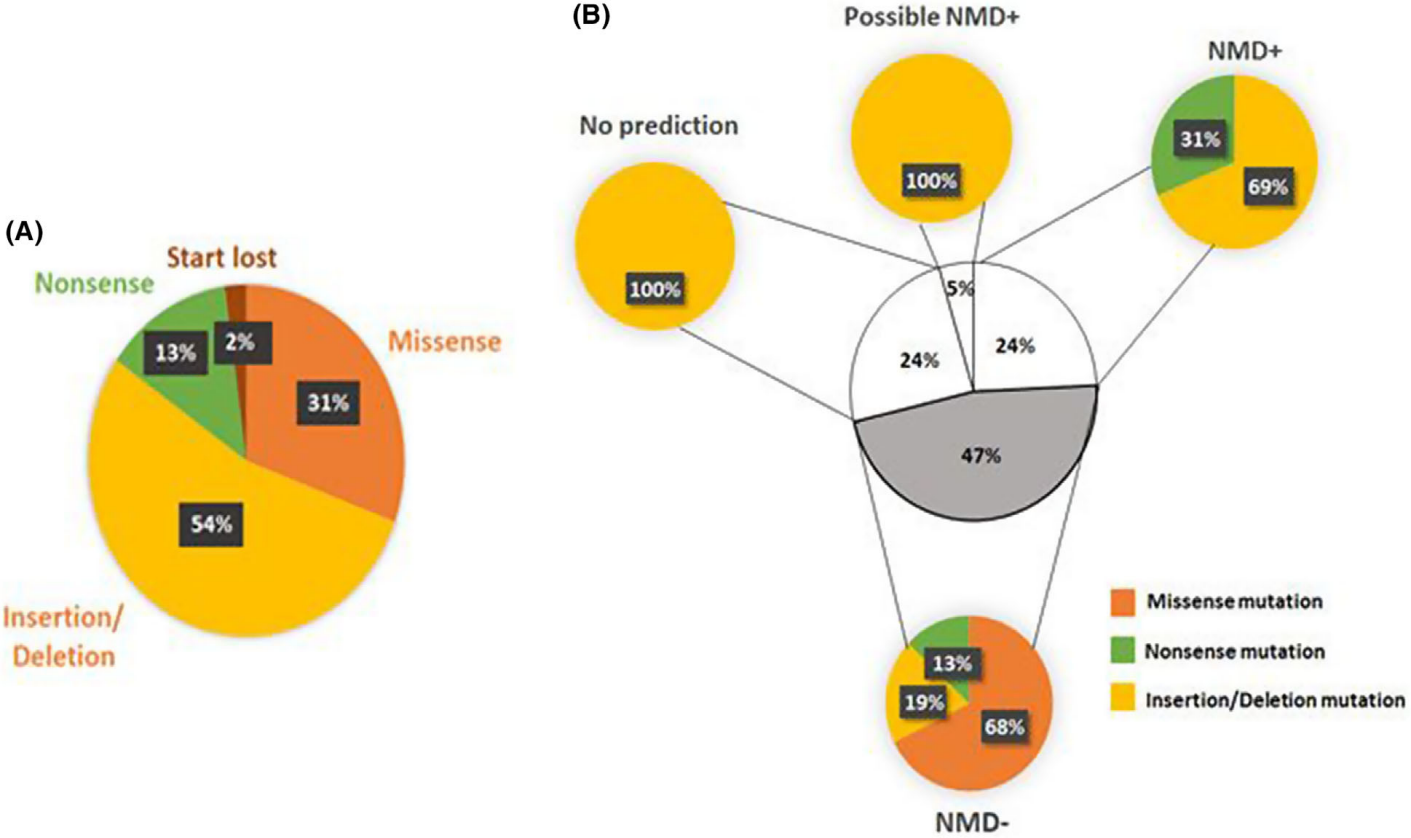
Mutation distribution and nonsense‐mediated mRNA degradation (NMD) analysis for RPS19 in Diamond Blackfan anemia (DBA). (A) distribution of mutation types reported in DBA patients. (B) NMD analysis of potential mRNA with DBA mutation. [Colour figure can be viewed at wileyonlinelibrary.com]

Based on the extent of RP‐ribosome possible interactions, we assumed that although the C‐terminal tail is an integral part of the protein, its truncation might be less harmful. Conversely, these mutations may result in a devastating event for the ribosome's maturation and/or function. These cases may result in a reduced number of ribosomes in the cells, in agreement with the results of genomic studies performed elsewhere [129]. In addition, a nonsense‐mediated mRNA decay (NMD) analysis [111] revealed that almost half of the mutated transcript variants (47%) might escape NMD (NMD−; Table S3 highlighted in green), whereas 29% undergo NMD (NMD+), possibly leading to protein depletion (Fig. 6). Notably, potential mRNA with DBA missense mutations escaped NMD, thus increasing the possibility of incorporating mutated protein in ribosome (Fig. 6B).

### eS19 pseudogene in DBA

Several ribosomal proteins are known to have pseudogenes [130, 131], and we analyzed the properties of seven of them [132]. An earlier report showed that pseudogenes *RPS19P1* and *RPS19P2*, which share 57% and 45% similarity with the wild‐type eS19, respectively, are not expressed [132], and a substantially higher similarity was obtained when the comparison was based on eS19 cDNA and pseudogene sequence [132]. In addition, a recent study has described an exciting suggestion of paralog‐switching in which canonical RPs are replaced by paralog RPs in *Drosophila* testis and ovary, expecting to alter the ribosome surface and regulate the process of translation. This study hints at the possibility of the incorporation of RP‐like proteins in the ribosome [133].

**Fig. 7:**
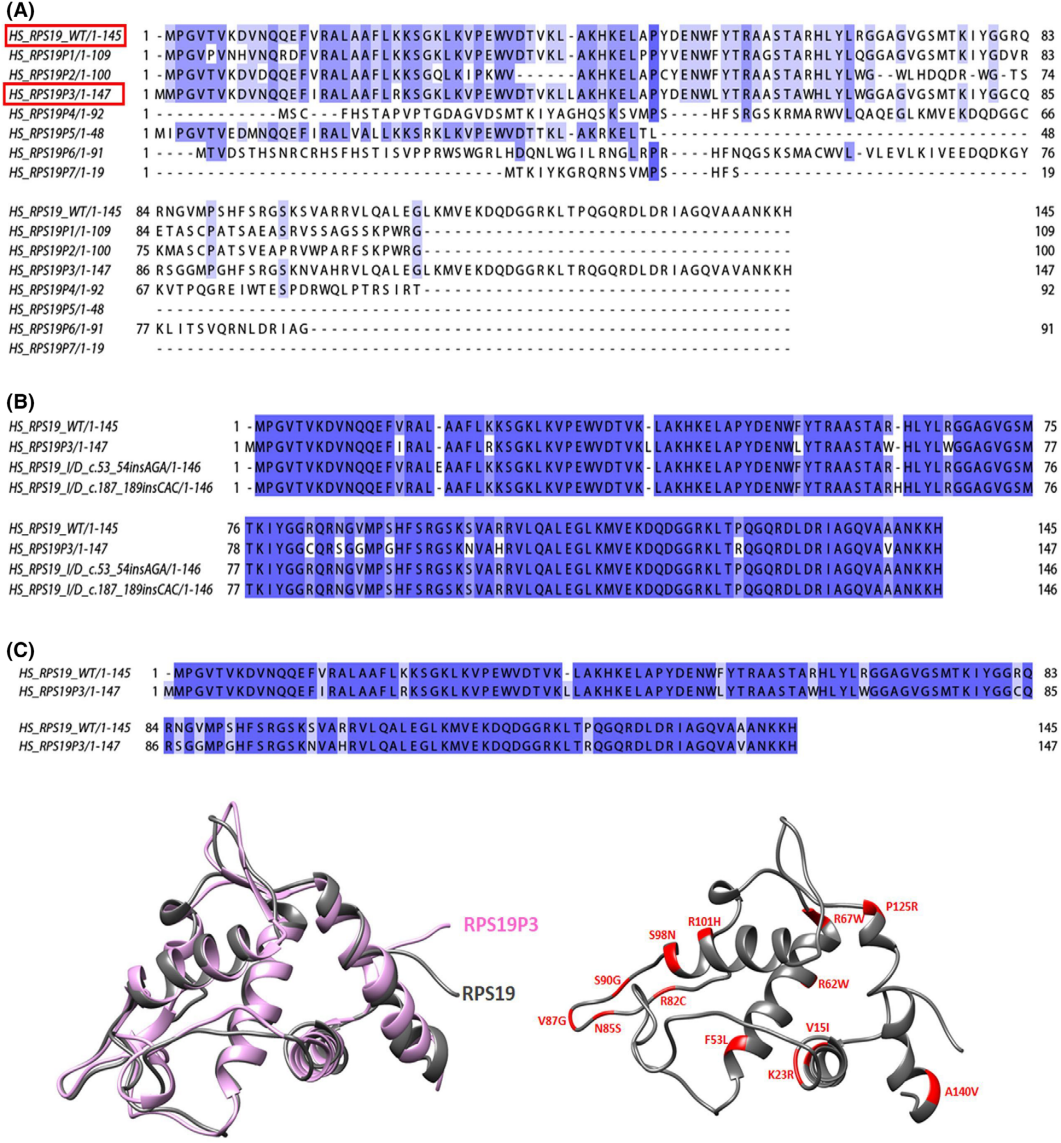
eS19 pseudogenes in Diamond Blackfan anemia. (A) Sequence alignment of eS19 with pseudogenes. The sequence of the wild‐type eS19 was aligned with its seven pseudogenes (RPS19P1, RPS19P2, RPS19P3, RPS19P4, RPS19P5, RPS19P6, RPS19P7) with clustalw [121] and visualized using jalview [122]. (B) Sequence alignment of RPS19P3 with predicted mutated protein eS19. Sequences of RPS19P3 were aligned with predicted mutated protein of c.53_54insAGA, (C)187_189insCAC RPS19 mutations with clustalw and visualized using jalview. (C) Pairwise alignment of eS19 wild‐type and the expected protein product of RPS19P3, left‐ the predicted structure of the protein product of RPS19P3 (pink) overlapped onto wild‐type eS19 (gray). Right‐ the differences in amino acid sequences are mapped onto the 3D structure of eS19, in red. [Colour figure can be viewed at wileyonlinelibrary.com]

There are no reported paralogs for *RPS19*; however, our prediction of potential protein sequences of the pseudogene revealed that one of them, *RPS19P3*, shares 90.4% identity and 93.2% similarity with the wild‐type eS19 (Fig. 7 and Table 1). In addition, the mutations that escape NMD share some homology with *RPS19P3* pseudogene (Fig. 7B). Notably, the amino acid differences between eS19 and *RPS19P3* coincide with DBA reported mutation, predicted structure of *RPS19P3* is similar to eS19 (Fig. 7C).

**Table 1:** Homology between *RPS19P3* pseudogenes and the expected mutated protein.

Pseudogenes	Identity (%)	Similarity (%)	Gaps (%)
RPS19P1	51.0	57.2	24.8
RPS19P2	41.5	45.3	45.9
RPS19P3	90.4	93.2	0.7
RPS19P4	31.4	35.8	50.9
RPS19P5	24.7	27.4	67.8
RPS19P6	20.6	26.3	65.1
RPS19P7	11.7	11.7	86.9

## Discussion and conclusions

In this study, we focused on the structure, location, and spatial positioning of protein eS19, the product of the ribosomal gene *RPS19*, which was suggested to participate in a regulatory process by binding its own mRNA in isolation [98], similar to a few other ribosomal proteins [99, 100, 101, 102, 103]. As this protein maintains the same overall specific 3D structure within the active ribosome and in isolation (Fig. 3), the preservation of this inherent 3D structure may indicate its importance and hints at functional conservation alongside comparable tasks.

Our detailed predictive analysis indicates various outcomes for the different eS19 mutations. Thus, we expect that although many eS19 mutations may not hamper ribosome biogenesis, those connected to functional relevance, for example, R62, may obstruct ribosomes assembly. Hence, our analyses led to the identification of nature's ingenious selective strategy to avoid ribosome function loss by impeding the biogenesis of ribosomes with a mutated member of its functional site. This new understanding of the relative functional and structural contributions sheds some light on the so far unexplained mechanism of *RPS19* involvement in impaired ribosome biogenesis. Moreover, the results of our analysis indicate the rather unanticipated finding that mutations, which are implicated mainly in dysregulation of ribosome biogenesis, may result from nature's attempts to avoid future ribosome malfunction. On the other hand, this distinctive procedure may allow the incorporation of eS19 with mutations located in positions away from the functional region, a point that has not yet been explored.

Owing to the assumption that some of the mutations that were identified in *RPS19* are expressed in eS19, we identified common denominators among the expected structural‐functional outcome of the various known *RPS19* DBA mutations. Importantly, these findings are supported by additional observations, mainly (a) the existence of functional ribosomes with truncated rRNA [134] and (b) the rRNA modification patterns that supersede temperature variations [135]. Thus, we revealed a natural mechanism for controlling ribosome biogenesis. We will not be surprised if similar associations will be detected in other cases related to ribosomopathies.

In principle, it seems that the studies reported here illuminate a fundamental aspect of natural mechanism to minimize or eliminate the incorporation of ribosomal proteins with mutated functional sites by hampering ribosome maturation. However, although this mechanism seems to avoid the creation of malfunctioning ribosomes as it is based on disrupting ribosome biogenesis, it can lead to a reduction in the number of ribosomes or ribosome insufficiency.

Our structural approach was accompanied by a genomic analysis, based on recent studies showing that some pseudogenes maintain or might have regained protein‐coding capacity [136], thus suggesting that pseudogenes may also contribute to the transcriptome and proteome of various species. Some pseudogenes are evolutionarily conserved [137], a property that may be a key to understanding unique disease subtypes [138] and tissue‐specificity [139]. This raises questions about the expression of pseudogenes of RPs in ribosomopathies. Spatiotemporal expression pattern and unique functions ascribed to pseudogenes of a few proteins, including PTEN, HTR7, and SUMO1 [140, 141, 142, 143, 144], may open a new direction for further investigations of ribosomopathies.

About a decade ago, DBA was identified as a ribosomal puzzle [145]. Since then, the understanding of the molecular basis of this disease underwent significant progress, yet many unanswered questions remain. We hope that our combined structural, bioinformatical, and genetic approach to elucidate phenotype–genotype correlations of genetic diseases creates an opening for subsequent consequent evaluation of additional questions relating to the connection between genetic modifications of components of human ribosomes and their expression in various ribosomopathies.

## Conflict of interest

The authors declare no conflict of interest.

## Author contributions

D‐GH, AR, and EZ performed the analyses, AY and AB designed the studies, HY conceived, initiated, and supervised the project, D‐GH, AB, AY, and HY wrote the manuscript with contributions and approval from all other authors.

## Supporting information


**Table S1.** Distribution of RPS19 mutations on its structural motifs.

**Table S2.** The Predicted interatomic contacts of eS19 atoms whose distances to their neighbors are less than or equal to 4 Å.

**Table S3.** Predicted eS19_NMD results (NM_001022.4).

**Fig. S1.** RPS19 missense mutation affecting rRNA environment in DBA.

**Fig. S2.** 40S intermediates & eS19. The figure is grouped based on the “State”, i.e., the actual 40S maturation stage, comparing the effects of mutations on the H bonds. The predicted H‐bonds are represented as blue dotted lines in both WT‐eS19 (red) and the mutated eS19 (pink with mutated residues in blue) at different stages of 40S maturation (called “states”, from A to D).

**Fig. S3.** RPS19 mutation map in DBA.

**Fig. S4.** RPS19 mutation effects the length of eS19 protein. Sequences of wild‐type eS19 were aligned with predicted mutated protein eS19 on different type of mutations using jalview [133] (a) Frameshift mutations and Nonsense mutations (b‐c) Insertion and Deletion mutations (d) Stop gained mutations and Missense mutations (e) Missense mutations.

## Data Availability

The data that support the findings of this study are available in the Supporting Information of this article.
