# Supplementary material for: Mutations in RPS19 may affect ribosome function and biogenesis in Diamond Blackfan anemia
Source: FEBS Open Bio. 2022 Jun 6;12(7):1419–34. doi: 10.1002/2211-5463.13444 (PMC9249338; doi:10.1002/2211-5463.13444)
Supplement: Supplementary file 1 — Table S1. Distribution of RPS19 mutations on its structural motifs. [file FEB4-12-1419-s007.docx]

**Supplementary Table S1. Distribution of RPS19 mutations on its structural motifs**

| **Structure** | **Mutations** | **Affected amino acid** |
| --- | --- | --- |
| **Helix** | **c.28_29insT**  **c.34_47del**  **c.36_37insAG**  **c.31C>T**  **c.34C>T**  **c.34_47del**  **c.36_37insAG**  **c.14_15insA**  **c.19del13bp**  **c.24del18bp**  **c.49G>C**  **c.33-34 GC > TT**  **c.29_30insA**  **c.33delG**  **c.43G>T**  **c.53T>C**  **c.53_54insAGA**  **c.58delG**  **c.58G>C**  **c.62T>C**  **c.154T>C**  **c.155G>A**  **c.156G>A**  **c.156G>C**  **c.166C>T**  **c.167G>A**  **c.169G>C**  **c.172G>C**  **c.173-2A>T**  **c.173-1delG**  **c.173-1G>A**  **c.176C>T**  **c.178A>C**  **c.182C>A**  **c.184C>T**  **c.185G>A**  **c.187_189insCAC**  **c.191T>C**  **c.195C>G**  **c.197_207del**  **c.212G>A**  **c.222delC**  **c.289_290insAGGC**  **c.295_296delGT**  **c.296_297delTG**  **c.301C>T**  **c.305G>C**  **c.307delG**  **c.320T>G**  **c.328delC**  **c.376C>T**  **c.380G>A**  **c.382C>T** | **N10, Q11, Q12, E13, V15, A17, L18, A20, F21**  **W52, R56, A57, A58, S59, T60, A61, R62, H63, L64, Y65, L66, R67,**  **G71, M75,**  **K97, V99, A100, R101, R102, V103, Q105, L107, L110,**  **Q126, Q128,** |
| **Loop** | **c.83T>G**  **c.88delG**  **c.93delC**  **c.98G>A**  **c.103dupG**  **c.104_105insA**  **c.106_107insA**  **c.112A>T**  **c.[134_135delinsAA;139_140insTC]**  **c.140C>T**  **c.144C>A**  **c.203_204insG**  **c.226A>C**  **c.233_250del**  **c.242_243insG**  **c.250_251delAG**  **c.250_251insA**  **c.274_304del**  **c.280C>T**  **c.281G>T**  **c.284delG**  **c.338_340delTGG**  **c.340G>T**  **c.344delA**  **c.344_345insAA**  **c.356_357insG**  **c.238_239insG**  **c.293_294delGT**  **c.329delG**  **c.341delA**  **c.357-1G>A**  **c.357-1G>T**  **c.358G>A**  **c.358G>C**  **c.372_373insA**  **c.384_385delAA**  **c.386_387ins8**  **c.390_391delTC**  **c.392T>C**  **c.392T>G** | **L28, V30, E32, W33, V34, E35, T36, L45, P47,Y48,**  **G69,**  **T76, I78, G81, R82, Q83, R84, H91, F92, R94,G95**  **M112, V113, E114, K115, D116, D118, G120,**  **D130, L131,** |
| **N-terminus** | **c.10_13delGTTA**  **c.13_14insA**  **c.14delC**  **c.20_32del**  **c.25_42del** | **V4, T5, V6, K7, V9,** |
| **C-terminus** | **c.401_402insT**  **c.403G>A**  **c.406 G>T**  **c.412delG**  **c.417delA**  **c.418delG**  **c.435_*3del** | **A135, G136, V138, A140, N142** |
