# Supplementary material for: Mutations in RPS19 may affect ribosome function and biogenesis in Diamond Blackfan anemia
Source: FEBS Open Bio. 2022 Jun 6;12(7):1419–34. doi: 10.1002/2211-5463.13444 (PMC9249338; doi:10.1002/2211-5463.13444)
Supplement: Supplementary file 3 — Table S3. Predicted eS19_NMD results (NM_001022.4). [file FEB4-12-1419-s004.docx]

**Ribosomal RNA/proteins interactions involved in Diamond Blackfan Anemia**

**Supplementary Table S3. Predicted eS19_NMD results (NM_001022.4)**

Transcripts that may escape non-sense mediated mRNA degradation (NMD-) are highlighted in green

**List of mutations observed in patients:**

| **Mutation** | **Exon/**  **intron** | **DNA mutation** | **NMD+/NMD-** | **Predicted**  **protein length** |
| --- | --- | --- | --- | --- |
| **Missense Mutation** |  |  |  |  |
| Missense mutation | Exon 2 | c.43G>T | NMD- | 145 |
| Missense mutation | Exon 2 | c.49G>C | NMD- | 145 |
| Missense mutation | Exon 2 | c.53T>C | NMD- | 145 |
| Missense mutation | Exon 2 | c.58G>C | NMD- | 145 |
| Missense mutation | Exon 2 | c.62T>C | NMD- | 145 |
| Missense mutation | Exon 3 | c.83T>G | NMD- | 145 |
| Missense mutation | Exon 3 | c.140C>T | NMD- | 145 |
| Missense mutation | Exon 3 | c.154T>C | NMD- | 145 |
| Missense mutation | Exon 3 | c.156G>C | NMD- | 145 |
| Missense mutation | Exon 3 | c.169G>C | NMD- | 145 |
| Missense mutation | Exon 3 | c.156G>T | NMD- | 145 |
| Missense mutation | Exon 3 | c.167G>A | NMD- | 145 |
| Missense mutation | Exon 3 | c.172G>C | NMD- | 145 |
| Missense mutation | Exon 3 | c.182C>A | NMD- | 145 |
| Missense mutation | Exon 3 | c.114G>C | NMD- | 145 |
| Missense mutation | Exon 4 | c.176C>T | NMD- | 145 |
| Missense mutation | Exon 4 | c.178A>C | NMD- | 145 |
| Missense mutation | Exon 4 | c.182C>A | NMD- | 145 |
| Missense mutation | Exon 4 | c.184C>T | NMD- | 145 |
| Missense mutation | Exon 4 | c.185G>A | NMD- | 145 |
| Missense mutation | Exon 4 | c.191T>C | NMD- | 145 |
| Missense mutation | Exon 4 | c.191T>G | NMD- | 145 |
| Missense mutation | Exon 4 | c.200G>C | NMD- | 145 |
| Missense mutation | Exon 4 | c.212G>A | NMD- | 145 |
| Missense mutation | Exon 4 | c.226A>C | NMD- | 145 |
| Missense mutation | Exon 4 | c.281G>T | NMD- | 145 |
| Missense mutation | Exon 4 | c.284G>T | NMD- | 145 |
| Missense mutation | Exon 4 | c.286T>C | NMD- | 145 |
| Missense mutation | Exon 4 | c.301C>T | NMD- | 145 |
| Missense mutation | Exon 4 | c.301C>G | NMD- | 145 |
| Missense mutation | Exon 4 | c.302G>A | NMD- | 145 |
| Missense mutation | Exon 4 | c.302G>C | NMD- | 145 |
| Missense mutation | Exon 4 | c.305G>C | NMD- | 145 |
| Missense mutation | Exon 4 | c.320T>G | NMD- | 145 |
| Missense mutation | Exon 4 | c.320T>A | NMD- | 145 |
| Missense mutation | Exon 4 | c.353A>G | NMD- | 145 |
| Missense mutation | Exon 5 | c.358G>A | NMD- | 145 |
| Missense mutation | Exon 5 | c.358G>C | NMD- | 145 |
| Missense mutation | Exon 5 | c.379G>A | NMD- | 145 |
| Missense mutation | Exon 5 | c.380G>A | NMD- | 145 |
| Missense mutation | Exon 5 | c.392T>C | NMD- | 145 |
| Missense mutation | Exon 5 | c.392T>G | NMD- | 145 |
| Missense mutation | Exon 5 | c.403G>A | NMD- | 145 |
|  |  |  |  |  |
| **Insertion /**  **Deletion Mutation** |  |  |  |  |
| Deletion (frameshift) | Exon 2 | c.10_13delGTTA | NMD+ | 4 |
| Insertion (frameshift) | Exon 2 | c.12_13insA | No prediction |  |
| Insertion (frameshift) | Exon 2 | c.13_14insA | No prediction |  |
| Deletion (frameshift) | Exon 2 | c.14delC | NMD+ | 5 |
| Insertion (frameshift) | Exon 2 | c.14_15insA | No prediction | 49 |
| Insertion (frameshift) | Exon 2 | c.14_15insT | No prediction | 49 |
| Deletion (frameshift) | Exon 2 | c.18delA | NMD+ | 8 |
| Deletion (frameshift) | Exon 2 | c.19del13bp | NMD+ | 23 |
| Deletion (frameshift) | Exon 2 | c.20_32del | NMD+ | 23 |
| Deletion | Exon 2 | c.24del18bp | NMD- | 139 |
| Deletion | Exon 2 | c.25_42del | NMD- | 139 |
| Insertion (frameshift) | Exon 2 | c.28_29insT | No prediction | 49 |
| Insertion (frameshift) | Exon 2 | c.29_30insA | No prediction | 49 |
| Deletion (frameshift) | Exon 2 | c.33delG | NMD+ | 27 |
| Insertion (frameshift) | Exon 2 | c.34_35insAG | NMD+ | 28 |
| Deletion (frameshift) | Exon 2 | c.34_47del | No prediction | 44 |
| Insertion (frameshift) | Exon 2 | c.36_37insAG | NMD+ | 44 |
| Insertion | Exon 2 | c.53_54insAGA | NMD- | 146 |
| Deletion (frameshift) | Exon 2 | c.58delG | NMD+ | 27 |
| Insertion/Deletion (frameshift) | Exon 3 | c.77_79del GGAinsTG | NMD+ | 27 |
| Deletion (frameshift) | Exon 3 | c.88delG | NMD+ | 74 |
| Deletion (frameshift) | Exon 3 | c.93delC | NMD+ | 74 |
| Insertion (frameshift) | Exon 3 | c.101_102insG | No prediction | 49 |
| Insertion (frameshift) | Exon 3 | c.103dupG | No prediction | 49 |
| Insertion (frameshift) | Exon 3 | c.104_105insA | No prediction | 49 |
| Insertion (frameshift) | Exon 3 | c.105_106insA | No prediction | 49 |
| Insertion (frameshift) | Exon 3 | c.106_107insA | No prediction | 49 |
| Insertion/Deletion (frameshift) | Exon 3 | [c.134_135delinsAA;  c.139_140insTC] | NMD+ | 75 |
| Insertion/Deletion (frameshift) | Exon 3 | TT>AA c.157-158, c.160insCT | NMD+ | 75 |
| Insertion | Exon 4 | c.187_189insCAC | NMD- | 146 |
| Insertion (frameshift) | Exon 4 | c.203_204insG | No prediction | 152 |
| Deletion (frameshift) | Exon 4 | c.196_206del11bp | No prediction | 148 |
| Deletion (frameshift) | Exon 4 | [c.197_207del](http://www.dbagenes.unito.it/variants.php?select_db=RPS19&action=view&view=0000117%2C0000046%2C0) | No prediction | 71 |
| Deletion (frameshift) | Exon 4 | [c.222delC](http://www.dbagenes.unito.it/variants.php?select_db=RPS19&action=view&view=0000118%2C0000047%2C0) | NMD+ | 74 |
| Deletion | Exon 4 | c.233_250del | NMD- | 146 |
| Insertion (frameshift) | Exon 4 | c.237 insG | No prediction | 152 |
| Insertion (frameshift) | Exon 4 | c.238_239insG | No prediction | 152 |
| Insertion (frameshift) | Exon 4 | c.242_243insG | No prediction | 152 |
| Deletion (frameshift) | Exon 4 | c.248_249delAG | No prediction | 151 |
| Deletion (frameshift) | Exon 4 | c.250_251delAG | No prediction | 151 |
| Insertion (frameshift) | Exon 4 | c.250_251insA | No prediction | 152 |
| Deletion (frameshift) | Exon 4 | c.273_303del CTTCAG  CCGAGTCCAAGAGTGTGGCCCGC | NMD+ | 99 |
| Deletion (frameshift) | Exon 4 | c.274_304del | NMD+ | 148 |
| Deletion (frameshift) | Exon 4 | c.284delG | NMD+ | 109 |
| Insertion (frameshift) | Exon 4 | c.289_290insAGGC | No prediction | 153 |
| Deletion (frameshift) | Exon 4 | 293_294delGT | No prediction | 151 |
| Deletion (frameshift) | Exon 4 | c.295_296delGT | No prediction | 151 |
| Deletion (frameshift) | Exon 4 | c.296_297delTG | No prediction | 151 |
| Deletion | Exon 4 | c.298_315del | NMD- | 145 |
| Deletion (frameshift) | Exon 4 | c.307delG | NMD+ | 109 |
| Deletion (frameshift) | Exon 4 | c.309 delG | NMD+ | 109 |
| Deletion (frameshift) | Exon 4 | c.328delC | NMD+ | 109 |
| Deletion (frameshift) | Exon 4 | c.329delG | NMD+ | 122 |
| Deletion (frameshift) | Exon 4 | c.331 delC | NMD- | 109 |
| Deletion (frameshift) | Exon 4 | c.336delG | NMD- | 122 |
| Deletion | Exon 4 | c.338_340delTGG | NMD- | 109 |
| Deletion (frameshift) | Exon 4 | c.341delA | NMD- | 122 |
| Deletion (frameshift) | Exon 4 | c.344delA | NMD- | 122 |
| Insertion (frameshift) | Exon 4 | c.344_345insAA | NMD- | 123 |
| Insertion (frameshift) | Exon 4 | c.347insC | No prediction | 152 |
| Insertion (frameshift) | Exon 5 | c.372_373insA | No prediction | 152 |
| Deletion (Frameshift) | Exon 5 | c.383_384delAA | No prediction | 151 |
| Deletion (frameshift) | Exon 5 | c.384_385delAA | No prediction | 151 |
| Deletion (Frameshift) | Exon 5 | c.385_386delAG | No prediction | 151 |
| Insertion (frameshift) | Exon 5 | c.386_387ins8 | Possible NMD | 288 |
| Deletion (frameshift) | Exon 5 | c.386_387 delGA | No prediction | 151 |
| Deletion (frameshift) | Exon 5 | c.390_391delTC | No prediction | 151 |
| Insertion (frameshift) | Exon 5 | c.401_402insT | Possible NMD | 152 |
| Deletion (frameshift) | Exon 6 | c.412delG | Possible NMD | 285 |
| Deletion (frameshift) | Exon 6 | c.417delA | Possible NMD | 285 |
| Deletion (frameshift) | Exon 6 | c.418delG | Possible NMD | 285 |
| Deletion | Exon 6 | c.435_*3del | NMD- | 145 |
| Insertion | Exon 6 | 434del7bp | Possible NMD | 145 |
|  |  |  |  |  |
|  |  |  |  |  |
| **Nonsense Mutations** |  |  |  |  |
| Nonsense mutation | Exon 1 | c.31C>T | NMD- | 10 |
| Nonsense mutation | Exon 1 | c.34C>T | NMD+ | 11 |
| Nonsense mutation | Exon 1 | c.33-34 GC > TT | NMD+ | 11 |
| Nonsense mutation | Exon 3 | c.94G>T | NMD- | 31 |
| Nonsense mutation | Exon 3 | c.98G>A | NMD- | 32 |
| Nonsense mutation | Exon 3 | c.99G>A | NMD- | 32 |
| Nonsense mutation | Exon 3 | c.112A>T | NMD- | 37 |
| Nonsense mutation | Exon 3 | c.144C>A | NMD+ | 47 |
| Nonsense mutation | Exon 3 | c.144C>G | NMD+ | 47 |
| Nonsense mutation | Exon 3 | c.155G>A | NMD+ | 51 |
| Nonsense mutation | Exon 3 | c.156G>A | NMD+ | 51 |
| Nonsense mutation | Exon 3 | c.166C>T | NMD+ | 55 |
| Nonsense mutation | Exon 3 | c.167G>A | NMD- | 145 |
| Nonsense mutation | Exon 4 | [c.195C>G](http://www.dbagenes.unito.it/variants.php?select_db=RPS19&action=view&view=0000199%2C0000111%2C0) | NMD- | 64 |
| Nonsense mutation | Exon 4 | [c.280C>T](http://www.dbagenes.unito.it/variants.php?select_db=RPS19&action=view&view=0000017%2C0000007%2C0) | NMD- | 93 |
| Nonsense mutation | Exon 4 | [c.340G>T](http://www.dbagenes.unito.it/variants.php?select_db=RPS19&action=view&view=0000027%2C0000008%2C0) | NMD+ | 113 |
| Nonsense mutation | Exon 5 | c.376C>T | NMD+ | 125 |
| Nonsense mutation | Exon 5 | c.382C>T | NMD+ | 127 |
|  |  |  |  |  |
|  |  |  |  |  |
| **Start Lost Mutations** |  |  |  |  |
| start lost | Exon 2 | c.1A>G |  | No protein |
| start lost | Exon 2 | c.3G>A |  | No protein |
| start lost | Exon 2 | c.3G>T |  | No protein |
|  |  |  |  |  |
|  |  |  |  |  |
