# Supplementary figures and images for: Mutations in RPS19 may affect ribosome function and biogenesis in Diamond Blackfan anemia
Source: FEBS Open Bio. 2022 Jun 6;12(7):1419–34. doi: 10.1002/2211-5463.13444 (PMC9249338; doi:10.1002/2211-5463.13444)

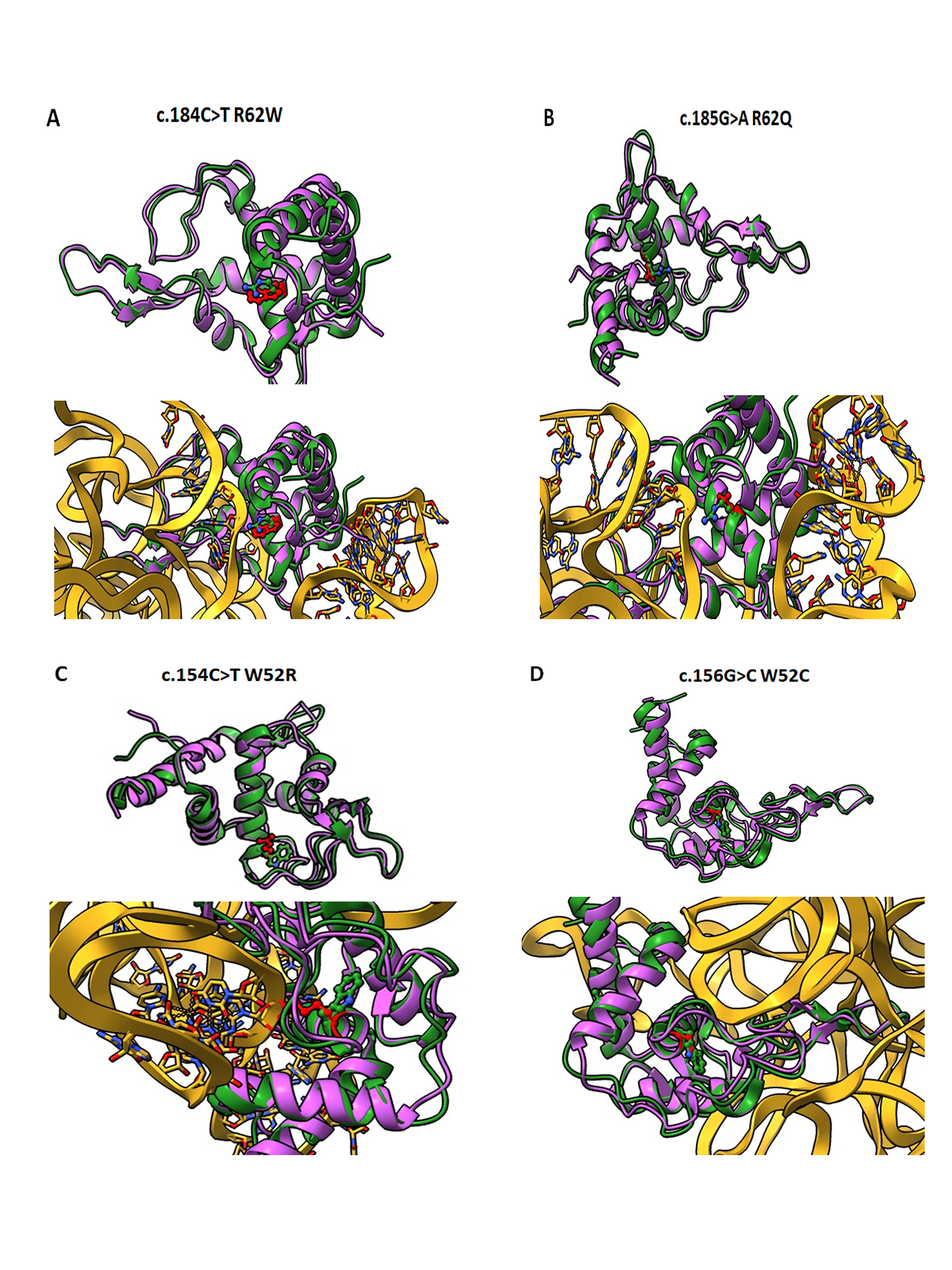

Supplement: Supplementary file 4 — Fig. S1. RPS19 missense mutation affecting rRNA environment in DBA. [file FEB4-12-1419-s005.jpg]

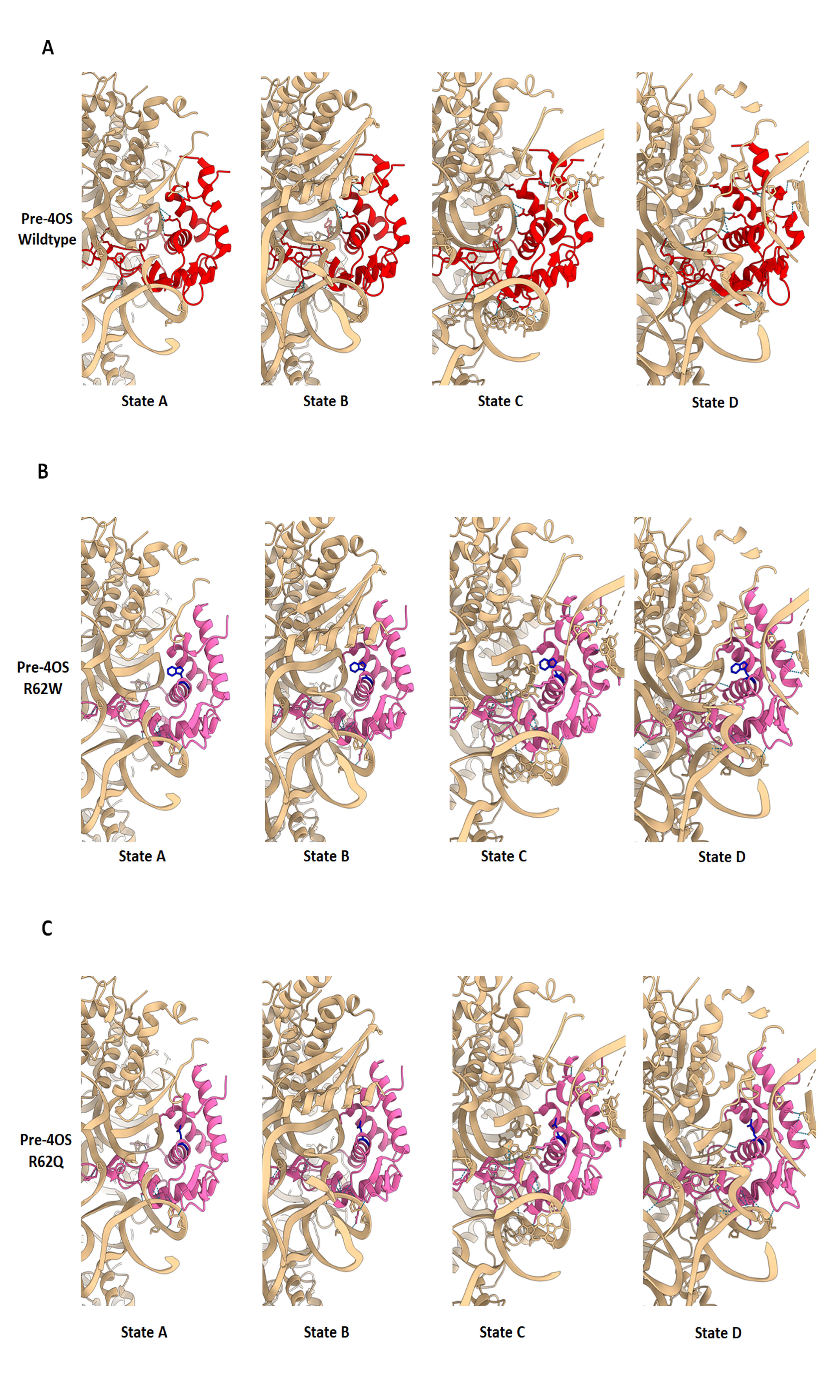

Supplement: Supplementary file 5 — Fig. S2. 40S intermediates & eS19. The figure is grouped based on the “State”, i.e., the actual 40S maturation stage, comparing the effects of mutations on the H bonds. The predicted H‐bonds are represented as blue dotted lines in both WT‐eS19 (red) and the mutated eS19 (pink with mutated residues in blue) at different stages of 40S maturation (called “states”, from A to D). [file FEB4-12-1419-s006.jpg]

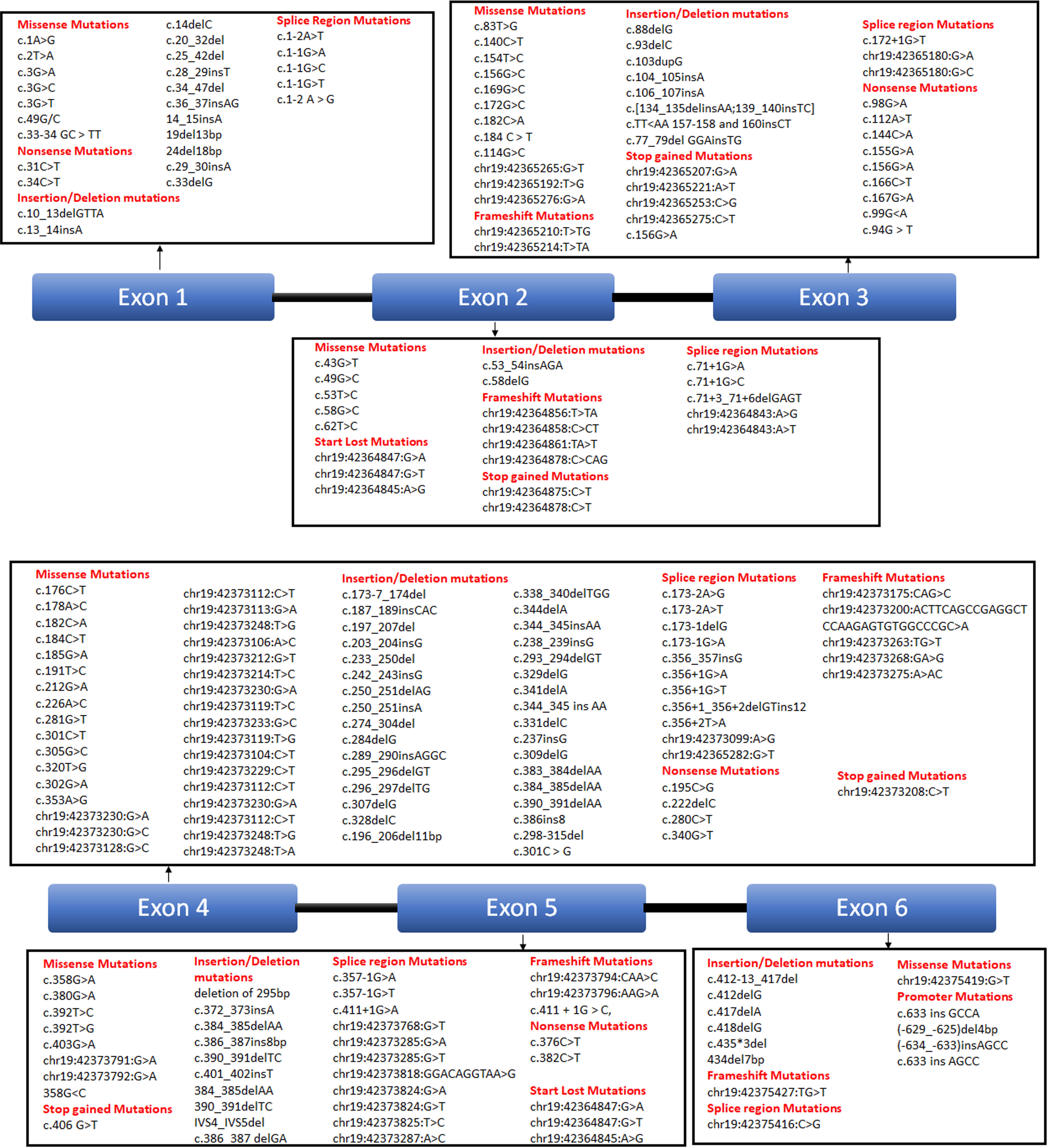

Supplement: Supplementary file 6 — Fig. S3. RPS19 mutation map in DBA. [file FEB4-12-1419-s002.jpg]

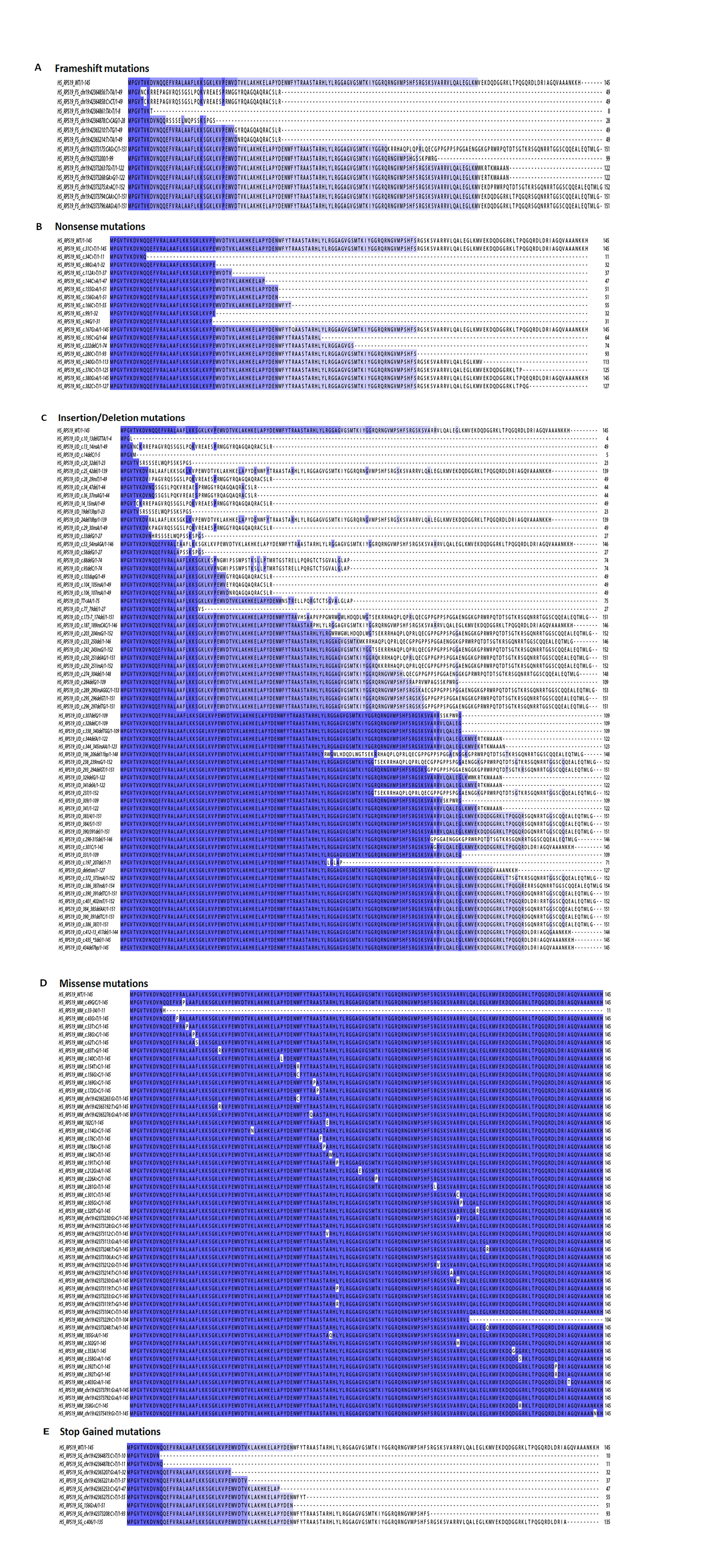

Supplement: Supplementary file 7 — Fig. S4. RPS19 mutation effects the length of eS19 protein. Sequences of wild‐type eS19 were aligned with predicted mutated protein eS19 on different type of mutations using jalview [133] (a) Frameshift mutations and Nonsense mutations (b‐c) Insertion and Deletion mutations (d) Stop gained mutations and Missense mutations (e) Missense mutations. [file FEB4-12-1419-s001.jpg]
